# Reprogramming natural proteins using unnatural amino acids

**DOI:** 10.1039/d1ra07028b

**Published:** 2021-11-26

**Authors:** Anup Adhikari, Bibek Raj Bhattarai, Ashika Aryal, Niru Thapa, Puja KC, Ashma Adhikari, Sushila Maharjan, Prem B. Chanda, Bishnu P. Regmi, Niranjan Parajuli

**Affiliations:** Biological Chemistry Lab, Central Department of Chemistry, Tribhuvan University Kritipur 44618 Kathmandu Nepal niranjan.parajuli@cdc.tu.edu.np; Department of Chemistry, Birendra Multiple Campus, Tribhuvan University Bharatpur Chitwan Nepal; Department of Chemistry and Physics, Southeastern Louisiana University Hammond Louisiana 70402 USA; Department of Chemistry, Florida Agricultural and Mechanical University Tallahassee Florida 32307 USA

## Abstract

Unnatural amino acids have gained significant attention in protein engineering and drug discovery as they allow the evolution of proteins with enhanced stability and activity. The incorporation of unnatural amino acids into proteins offers a rational approach to engineer enzymes for designing efficient biocatalysts that exhibit versatile physicochemical properties and biological functions. This review highlights the biological and synthetic routes of unnatural amino acids to yield a modified protein with altered functionality and their incorporation methods. Unnatural amino acids offer a wide array of applications such as antibody-drug conjugates, probes for change in protein conformation and structure–activity relationships, peptide-based imaging, antimicrobial activities, *etc.* Besides their emerging applications in fundamental and applied science, systemic research is necessary to explore unnatural amino acids with novel side chains that can address the limitations of natural amino acids.

## Abstract 2

Incorporation of unnatural amino acids into protein offers wide array of applications in fundamental and applied science.

## Graphical abstract

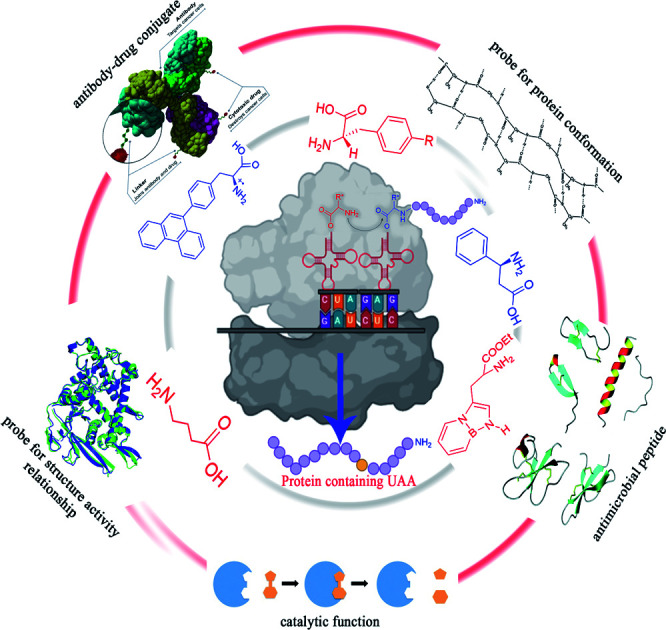

## 1. Introduction

A vast majority of the physiological processes of cells require proteins that catalyse biological reactions, regulate gene expression and the immune response, provide a structural framework, and modulate transport systems, among other things.^1^ Most surprisingly, proteins that perform these diverse functions are composed of 20 building blocks—the natural amino acids. Unnatural amino acids (UAAs) are synthesized and can be incorporated into proteins to expand the domain of protein functions.^2^ UAAs are not genetically encoded by organisms.^3^ Since UAAs are not present in natural polypeptide chains,^4^ they are also called non-proteinogenic or non-canonical amino acids (ncAA).

The structure of UAAs may resemble or differ significantly from natural amino acids and they are referred to as analogues or surrogates, respectively.^3^ The synthesis and applications of UAAs have received considerable attention, particularly in the field of enzymology and drug discovery. In protein engineering, the ability to substitute any natural amino acid with UAA at a particular point has emerged as a valuable molecular tool because such substitution allows the introduction of altered physicochemical and biological properties.^5^ UAAs can be incorporated into many structural units to develop potential leads in peptidic and non-peptidic complexes. The incorporation of UAAs into protein expands its functional diversity in biophysics, spectroscopy and optical probes, bio-orthogonal chemistry and protein labelling, post-translational modifications; their mimetics, signal transduction, protein interactions mapping, and photoactivated motifs.^6^ Furthermore, UAAs dramatically expand the possibilities for using chiral building blocks and molecular scaffolds to construct combinatorial libraries.^7^

UAAs can be synthesized chemically or they are produced naturally as secondary metabolites in several organisms, such as bacteria, fungi, plants, or marine organisms.^8^ Over the past few years, by constructing engineered microbial strains, various metabolic pathways have aided in the production of UAAs. Modern discoveries allow promising methods to produce sufficient amounts of proteins *in vitro* and *in vivo* using UAAs. The site-specific incorporation of UAAs into enzymes has enabled structural modification, accepting a wide range of substrates such as ketone, azide, alkyne, alkene, tetrazine, *etc.* Various probes, designed using UAAs, can be used for diverse applications, such as evaluating the effects of small molecule proteasome stimulators in live cells and comparing proteasome activity in different cancer cell types.^9^ Here, in this review, we have focused on the synthesis, incorporation approaches, applications of UAAs, as well as their limitations and prospects. We believe this review would provide useful information that helps researchers to reprogram natural proteins by incorporating UAAs.

## 2. Synthesis of UAAs

### 2.1 Biological synthesis

#### 2.1.1 Use of engineered bacterial strains

**Fig. 1:**
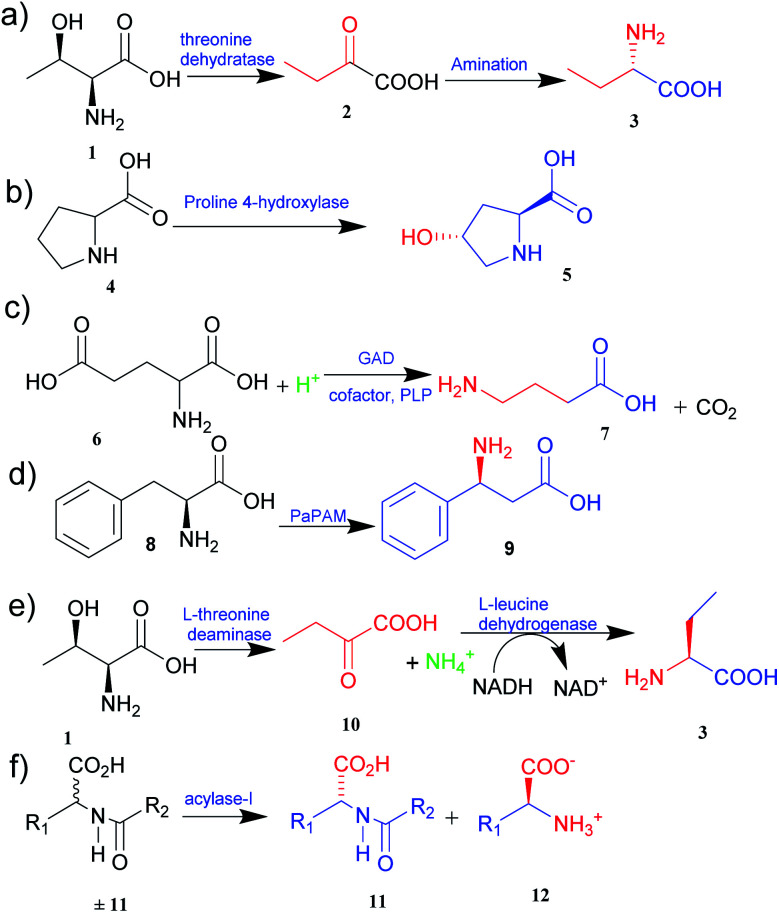
Synthesis of UAAs (a) synthesis of l-homoalanine 3 (b) synthesis of *trans*-4-hydroxy-l-proline 5 (c) synthesis of GABA 7 (d) synthesis of β-phenylalanine 9 (e) biosynthesis of L-ABA 3 (f) acylase-I for kinetic resolution of UAAs.

The metabolic engineering approach allows the creation of new pathways, reengineering of the existing ones, and the customization of enzyme activity to a specific purpose. Engineered microorganisms are used to synthesize various essential novel products, including UAAs.^10^ Zhang and co-workers^11^ expanded the natural metabolic capability and biosynthesized l-homoalanine directly from glucose using engineered *E. coli*. Threonine 1 synthesized after glycolysis was converted to 2-ketobutyrate 2 by threonine dehydratase and thus obtained 2-ketobutyrate was diverted to synthesize l-homoalanine 3 (Fig. 1a).^11^ Likewise, Xu *et al.*^12^ expanded the natural metabolic network of *E. coli* by coexpressing the *ilvA* gene from *E. coli* and *leuDH* gene from *Thermoactinomyces intermedius* to produce l-2-aminobutyric acid (l-ABA) directly from glucose. Based on the natural proline pathway, *trans*-4-hydroxy-l-proline (Hyp) 5 has been synthesized by constructing Hyp producing *E. coli* recombinant strains through the introduction of a proline 4-hydroxylase gene in an l-proline 4 producing *E. coli* (Fig. 1b).^13^ Similarly, γ-aminobutyric acid (GABA) 7 was known to be produced from decarboxylation of glutamic acid 6.^11^ Considering this fact, the glutamate decarboxylase, *LbGAD* found in *Lactobacillus brevis*, has been successfully engineered in *Bacillus subtilis* to develop a strain with an enhanced GABA productivity (Fig. 1c).^14^ By using similar strategies, various UAAs, such as l-azidohomoalanine,^15^ β-phenylalanine 9 (from phenylalanine 8) (Fig. 1d),^16^ norleucine,^17^ and several others, have been successfully biosynthesized. The success of these strategies has evoked researchers to investigate further metabolic pathways for the synthesis of pharmaceutically valuable UAAs.

#### 2.1.2 Biocatalytic synthesis

There are various possible routes to synthesize UAAs using enzymes. However, concerns with enzyme stability and substrate range, particularly with non-natural substrates, remain a barrier.^3^ These limitations can be mitigated by protein engineering, a powerful method for modifying amino acids and conformation to produce desirable and more effective catalysts.^18^

The two primary biocatalytic methods for the synthesis of UAAs are kinetic resolution and asymmetric synthesis. While the kinetic resolution is limited by a maximum theoretical yield of 50%, asymmetric synthesis provides a theoretical yield of 100%.^19,20^

Asymmetric synthesis is an effective process that occurs without any protective group(s) and is based on straightforward reaction steps to synthesize new UAAs. Substrates are asymmetrically converted into their optically pure form by using transaminase and dehydrogenase.^21^ Deamination of l-threonine 1 followed by hydrogenation gives a better theoretical yield than other processes (Fig. 1e).^22^ Various amino acid dehydrogenases have been developed and utilized for enzymatic chiral amino acid synthesis.^20,23^ Even naturally occurring amine dehydrogenases have been engineered to improve their substrate specificity and catalytic efficiency. Cai *et al.*^24^ have synthesized γ-aminobutyric acid from biobased lignocellulosic waste using an engineered amine dehydrogenase.

Similarly, asymmetric synthesis by transaminase is much simpler and effective since the substrates like keto acids can easily be converted into amino acids. Displaying broad substrate specificities, transaminase also plays a vital role in nitrogen metabolism as an industrial biocatalyst. It exhibits a rapid reaction rate and requires no external cofactor regeneration. Hence, α-transaminases like tyrosine, aspartate, valine–alanine, branched-chain, and aromatic amino acid transaminases are widely studied.^25^ Approximately 50% of UAAs successfully incorporated into proteins are derived from tyrosine or phenylalanine.^26^ Synthetically easily accessible α-keto acid is taken as a target intermediate that can be easily converted into an amino acid with the help of enzymes in a single step. The keto group is replaced with an amino group in a stereospecific manner. In this process, aminotransferase (transaminase), which uses pyridoxal 5′-phosphate as a cofactor, reversibly catalyses the transamination reaction.^26^ The transaminases possessing relaxed substrate specificity and high enantioselectivity are widely employed for the biosynthesis of many UAAs. l-Homophenylalanine was synthesized using an enzyme of transaminase class, namely aspartate aminotransferase isolated from *Paracoccus denitrifican.*^27^ Similarly, several l-thienylalanines^28^ and l-phosphinothricin^29^ have been synthesized by employing tyrosine aminotransferase.

The biological synthesis of UAAs by the kinetic resolution uses different enzymes such as lipases and nitrilases during the synthetic process.^30^ Acylase I (aminoacylase; *N*-acyl amino acids amidohydrolase), the most widely used and applicable enzymatic catalyst, has been employed for the kinetic resolution of UAAs and α-amino acids. This enzyme accepts the substrate with a broad range of functionality and structure. Chenault *et al.*^31^ have studied this approach to synthesize UAAs by utilizing acylase from *Aspergillus oryzae* and porcine kidney. In this method, acylase-I catalysed the enantioselective hydrolysis of *N*-acyl-l-amino acids ±11 to 11 and 12. d-Amino acid and l-amino acid products having a variety of uses were obtained with high enantiomeric excess (Fig. 1f).

#### 2.1.3 Protein engineering approach

Some challenges with enzymes such as low activity, low stability, and weak substrate range may be encountered during the synthesis of UAAs. Also, the tolerance of enzymes to a new substrate produced through metabolic engineering is limited. These shortcomings could be improved by using a protein engineering approach which includes rational design and directed evolution. Rational design predicts structural alterations required to achieve desired changes in a protein's property using molecular modelling, structural, and mechanistic knowledge. The difficulty of formulating valid predictions, on the other hand, led to directed evolution, which entails creating a large number of protein variations by random mutations and screening or selecting for those with the required traits. A combination of rational design and directed evolution has often been utilized to develop proteins with improved functions. The protein engineering approach introducing the enzymes belonging to diverse families of ammonia lyases and aminomutases has also expanded the limited use of specific enzyme classes like proteases, lipases, dehydrogenases, aldoses, nitrilases, transaminases, and others, contributing to UAAs synthesis.^32^ There are still surfeit pathways and enzymes to be unravelled for the further expedition of enormous useful UAAs. Thus, the future advancement in synthetic biology and bioinformatics would undoubtedly lead to further exploration of metabolic pathways and enzymes contributing to the synthesis of other useful UAAs.

### 2.2 Chemical synthesis

**Fig. 2:**
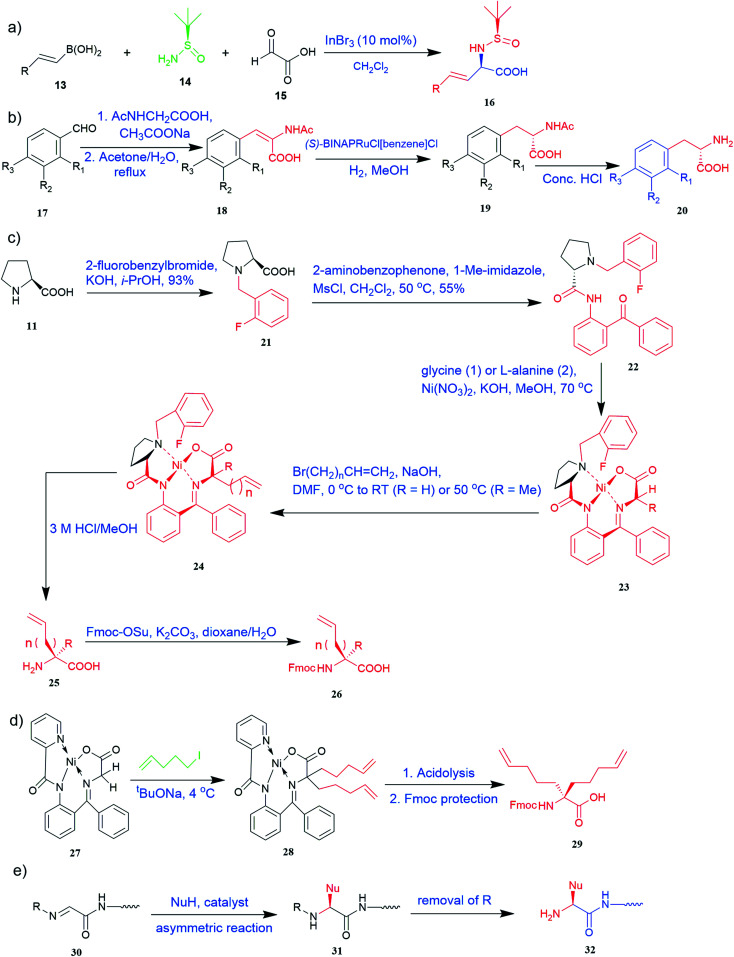
Synthesis of UAAs (a) Petasis borono–Mannich access to UAA 16 (b) synthesis from *N*-acetylamino phenyl acrylic acid 20 (c) synthetic route for unnatural alkenyl amino acids (25, 26) (d) synthesis of α-bisalkenyl substituted glycine 29 (e) asymmetric synthesis of UAA containing peptide 32.

**Fig. 3:**
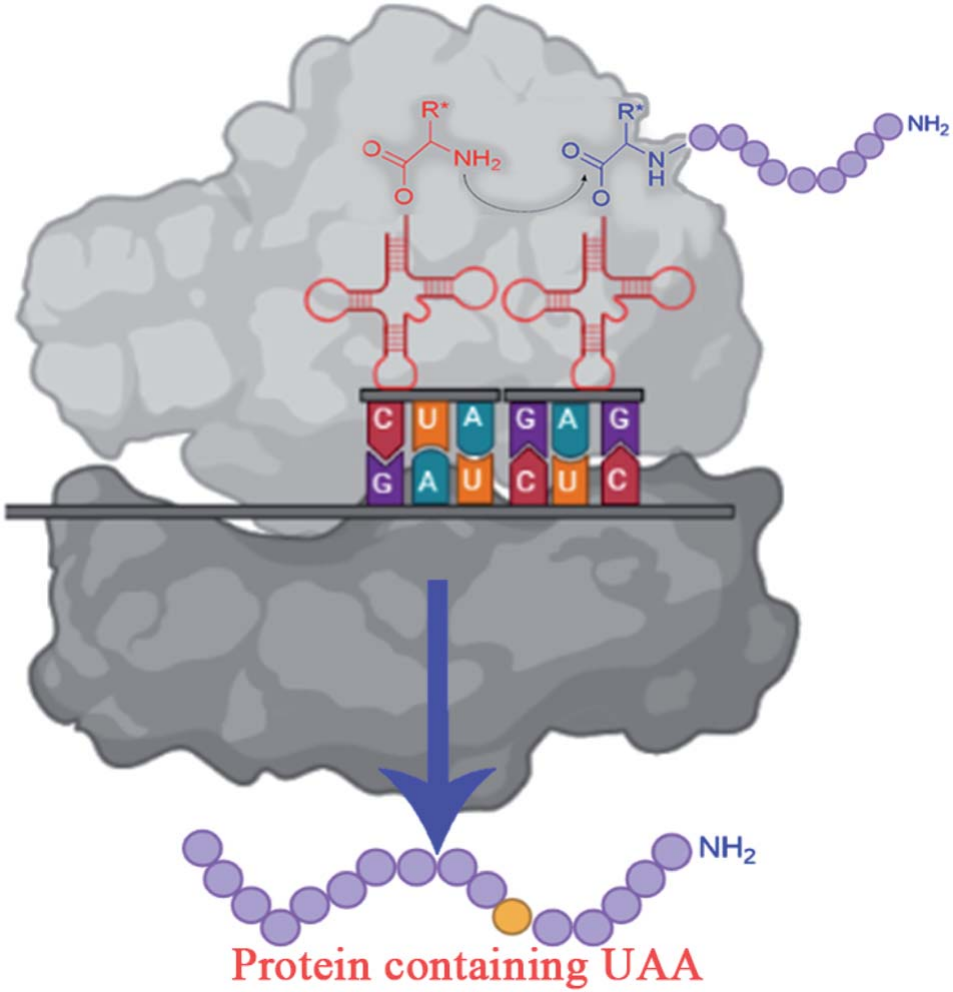
Incorporating unnatural amino acids into proteins using nonsense codon suppression.

**Fig. 4:**
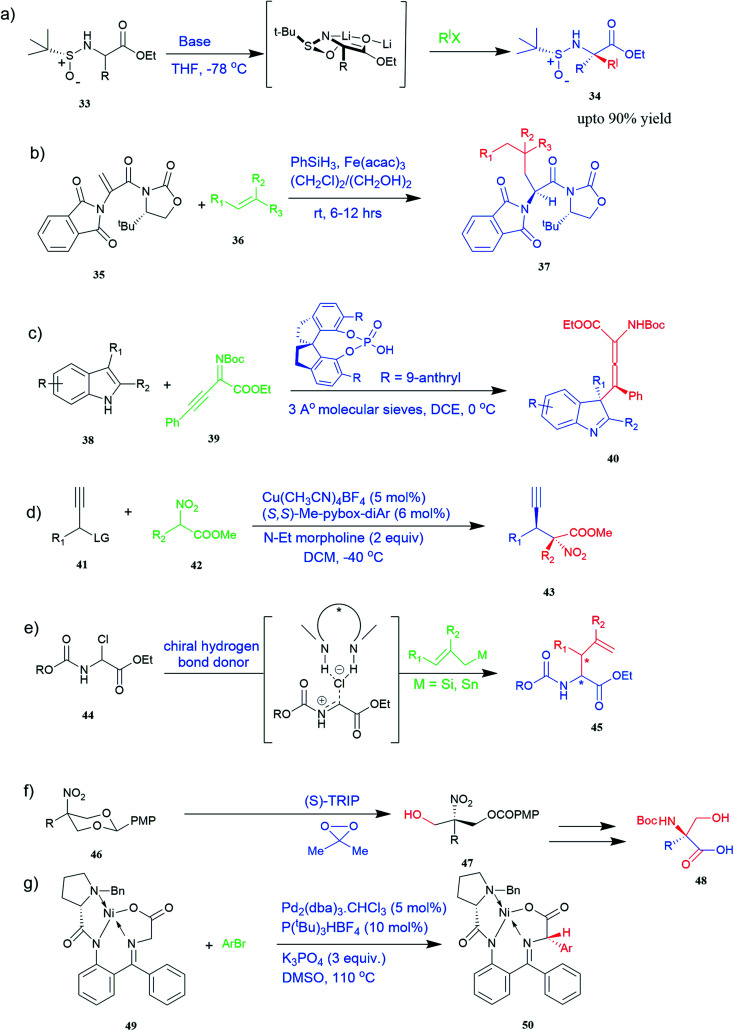
Synthesis of UAAs (a) diastereoselective synthesis of UAA by α-*tert*-butanesulfinamide 33 (b) iron-catalysed diastereoselective synthesis of unnatural chiral (*S*)-α-amino acid 37 (c) synthesis of tetrasubstituted α-amino allenoate 40 (d) synthesis of quaternary α-amino acid 43 (e) synthesis of α-allyl amino ester 45 (f) synthesis of unnatural α-substituted serine 48 (g) synthesis of arylglycine derivative 50.

**Fig. 5:**
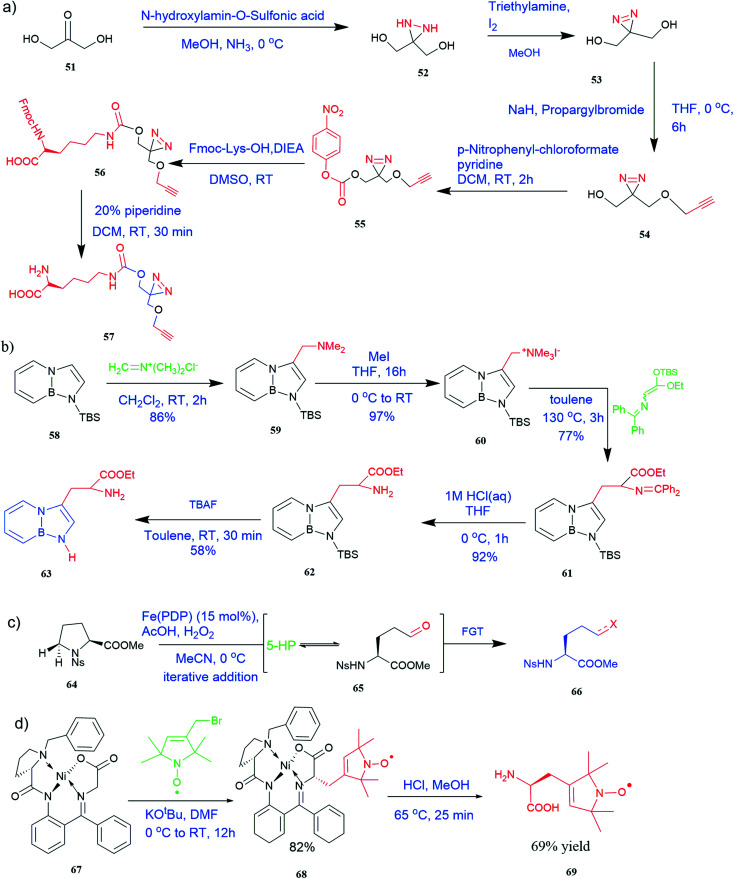
Synthesis of UAAs (a) synthesis of PrDiAzk 57 (b) synthesis of BN-tryptophan ester 63 (c) synthesis of UAA from oxidation of proline (d) synthesis of chiral spin-labeled amino acid 69.

Various synthetic methods have been developed to gain access to amino acid-based structures. Chemical routes are more profound than biocatalytic routes, possibly due to versatile synthetic ways. For the preparation of UAAs, a variety of chemical synthesis methods are available (Fig. 2–5).

#### 2.2.1 Asymmetric synthesis

The most efficient and effective way to produce all kinds of optically enriched α-amino acids is asymmetric catalytic synthesis, which plays an important role in the chemical production of pharmaceuticals. The highly diastereoselective Lewis acid-promoted Petasis three-component-reaction of *N-tert*-butanesulfinamide 14 was used to create a method for the asymmetric synthesis of β,γ-unsaturated α-amino acids^33^16 by conducting the reaction of glyoxylic acid 15 and vinyl boronic acid 13. The reaction was performed at room temperature to increase diastereoselectivity and yield. The optimized conditions included Lewis acid (InBr_3_), solvent (CH_2_Cl_2_), concentration (0.3 M), and time (12 h) (Fig. 2a).^34^

Of specific interest are catalytic methods with high levels of enantioselective control. A ruthenium catalyst was used for asymmetric hydrogenation of *N*-acetylamino phenyl acrylic acids 18 to yield chiral acids 20 (Fig. 2b), which are expected to be important in pharmaceuticals.^34,35^ Conformationally constrained peptides have a lower penalty for folding, which facilitates bioactive conformation. A new generation of chemical and drug discovery tools has emerged from conformationally constrained peptides. Asymmetric alkylation of a fluorine-modified Ni(ii) Schiff base complex (23, 24) yielded an unnatural alkenyl amino acid (25, 26) required for peptide ‘stapling’ (Fig. 2c).^36^ The most apparent technique for producing UAAs is the alkylation of glycine.^34^ Moving to a more economical and environmentally suitable chiral phase transfer catalyst, the asymmetric alkylation of glycine has a yield between 60% and 90%. However, these synthetic routes depend on toxic reagents such as methylsulfonyl chloride.^37,38^ To minimize the effect of toxic reagents, 1-ethyl-3-(3-dimethyl aminopropyl)carbodiimide/4-dimethylaminopyridine (EDCI/DMAP) as a coupling agent, a chiral intermediate *i.e.* derivative of glycine non-proline 27 was synthesized starting with proline and then alkylating in the presence of sodium *tert*-butoxide which obtained 28 (Fig. 2d). By using this method, five UAAs for peptide stapling 29 were prepared with high enantioselectivity >99% ee and yields ranging from 60% to 70%.^37^ Furthermore, a new methodology for the asymmetric synthesis of UAAs-containing peptides 32 was also developed using imino peptides 30 such as α-imino perfluro alkylesters, imides, or thioesters (Fig. 2e).^39^

#### 2.2.2 Diastereoselective synthesis

There are currently limited methods for producing α,α-disubstituted amino acids. New diastereoselective synthesis for the production of UAAs is a vital area of research in organic synthesis. Dwulet *et al.*^40^ devised a method for diastereoselective synthesis of UAAs by alkylation of α-*tert*-butanesulfinamide 33 auxiliary-bound enolates where chiral α-sulfinamido esters were alkylated under primary conditions, yielding up to 90% of mono and α,α-disubstituted amino acid derivatives 34 (Fig. 4a).^40^

Known as an inexpensive and abundant metal, iron is commonly utilized as a catalyst in organic synthesis. Developing an effective and practical diastereoselective method to synthesize unnatural chiral α-amino acids using iron as a catalyst is highly desired. Toward this end, an iron-catalysed diastereoselective method has been developed; the method employs widely accessible iron salts, 2-phthaloyl acrylamide 35, and alkenes 36 as starting materials, and phenyl silanes as a reductant for unnatural chiral (*S*)-α-amino acids with γ-quaternary carbon centres 37 (Fig. 4b).^41^ This protocol has several benefits, including simple and broad substrates, moderate conditions, excellent diastereoselectivity, and simple workup methods.

#### 2.2.3 Enantioselective synthesis

These days, there has been immense interest in the synthesis of enantioenriched allenic compounds; thus, several strategies have been developed to synthesize these compounds.^42–45^ In a method, dearomative γ-addition of 2,3-disubstituted indoles 38 to β,γ-alkynyl-α-imino esters 39 catalysed by chiral phosphoric acid was used to perform organocatalytic enantioselective synthesis of tetrasubstituted α-amino allenoates 40 (Fig. 4c).^42^ Zhu and colleagues found a Cu–pybox complex catalysed diastereo- and enantioselective propargylic substitution method employing propargylic carbonates 41 and α-substituted nitroacetates 42. This method produced a high yield of non-proteinogenic quaternary α-amino acid precursors 43 with two stereogenic centres and a terminal alkyne moiety (Fig. 4d).^43^ Bendelsmith *et al.*^44^ developed a method for enantioselective synthesis of α-allyl amino esters 45 which was achieved through allylation of α-chloro glycine esters 44 with a chiral squaramide hydrogen-bond donor anion-abstraction catalyst. Using allylsilane, fifteen α-allyl amino esters were synthesized with high enantioselectivity (up to 97% ee) (Fig. 4e).^44^ In addition, chiral phosphoric acid-mediated oxidative enantioselective desymmetrization of 2-substituted, 2-nitro-1,3-diolbenzylidine acetals 46 in the presence of dimethyldioxirane as an oxidizing agent resulted in the highly enantioselective synthesis of acyclic α-tertiary amines by asymmetric desymmetrization. This method allows for the formation of chiral 2-nitro-1,3-diols with high enantioselectivity, which could be transformed into optically pure, unnatural α-alkyl serine 48 (Fig. 4f).^45^

#### 2.2.4 Catalytic synthesis

Substituted aryl glycines are structural motifs in many biologically active compounds and natural products, such as peptide antibiotics, antiplatelet agents, and hepatitis C virus NS3/4A protease inhibitors. Numerous approaches for synthesizing acyl glycines have been developed, but only limited asymmetric approaches have been reported.^46^ Unlike biocatalysts which most commonly display enantioselectivity, prochiral substrates involving organic and inorganic catalysts rarely exhibit enantioselectivity. Zhang and colleagues demonstrated a stereoselective synthesis of optically active aryl glycine derivatives 49 utilizing a Pd-catalysed alpha arylation of chiral nickel(ii) glycinate complex 50 with different aryl bromides with up to 80% yield (Fig. 4g).

It has been demonstrated that bifunctional UAAs called PrDiAzK 57 can be inserted into the protein interface, using genetic code expansion, with a minimum structural perturbation. Hoffmann *et al.*^47^ demonstrated bifunctionality for UAAs based on *Z*-lysine, benzophenone-alkyne “BPKyne,” and γ-selenolysine, in which a lysine-based bifunctional amino acid combines a photoreactive diazirine group with a terminal alkyne handle for reaction with azides using copper-catalysed click chemistry. The starting material was commercially available dihydroxyacetone 51. In liquid ammonia, the ketone was converted into diazirine (52, 53) in a two-step reaction. Propargyl bromide was used to alkylate one of the two hydroxyl groups. The single-substituted product 54 was isolated, converted into a reactive carbonate, and bound to lysine (56, 57) (Fig. 5a).^47^ In the hope to utilize this UAA in further protein studies and classify its properties concerning the natural substrate, boron and nitrogen-containing unnatural analogues of tryptophan 63 have also been synthesized through the functionalization of BN-indole 58. In this case, TBS-BN-indole 58 was subjected to a substitution reaction with dimethyliminium chloride. Iodomethane was used to methylate the resulting product 59, which was then displaced with silyl-ketene-acetal. The Schiff base protecting group of 62 can be removed in aqueous acidic conditions. Following this, the silyl protecting group was deprotected to yield BN-tryptophan ethyl ester 63 (Fig. 5b).^48^ More notably, these studies demonstrated the first example of an azaborine containing amino acid being introduced into proteins.

#### 2.2.5 Chiral pool synthesis

Chiral pool synthesis is a strategy to enhance chiral synthesis efficiency, and this approach can extensively increase the production of UAAs. Osberger and his team reported a method that employed iron catalysts for the targeted C–H oxidative alteration of amino acids and peptides with the preservation of α-centre chirality.^49^ In this method, substituted proline 64 oxidation to 5-hydroxyproline furnishes an intermediate 65 with a highly versatile hemiaminal functional group that can be modified to UAAs and UAA-containing peptides 66 (Fig. 5c).^49^ In another approach, chiral spin-labelled amino acids 69 obtained by cleavage from their respective complexes (Belokon complex) 67 were added to d-amino acid oxidase (DAAO) to enhance enantiopurity. The d-amino acids were selectively transformed into α-keto acids, which can then be isolated from the desired l-amino acid using this catalytic procedure (Fig. 5d).^50^ In defiance of ecologically unfriendly nature, chemical synthesis is undeniably a non-tedious and robust technique for UAAs synthesis. Henceforth, a minute alternation in chemical procedure with green chemistry would nullify its toxic effects and assuredly be a novel synthetic method for UAAs.

### 2.3 Other methods

#### 2.3.1 Photochemical synthesis

**Fig. 6:**
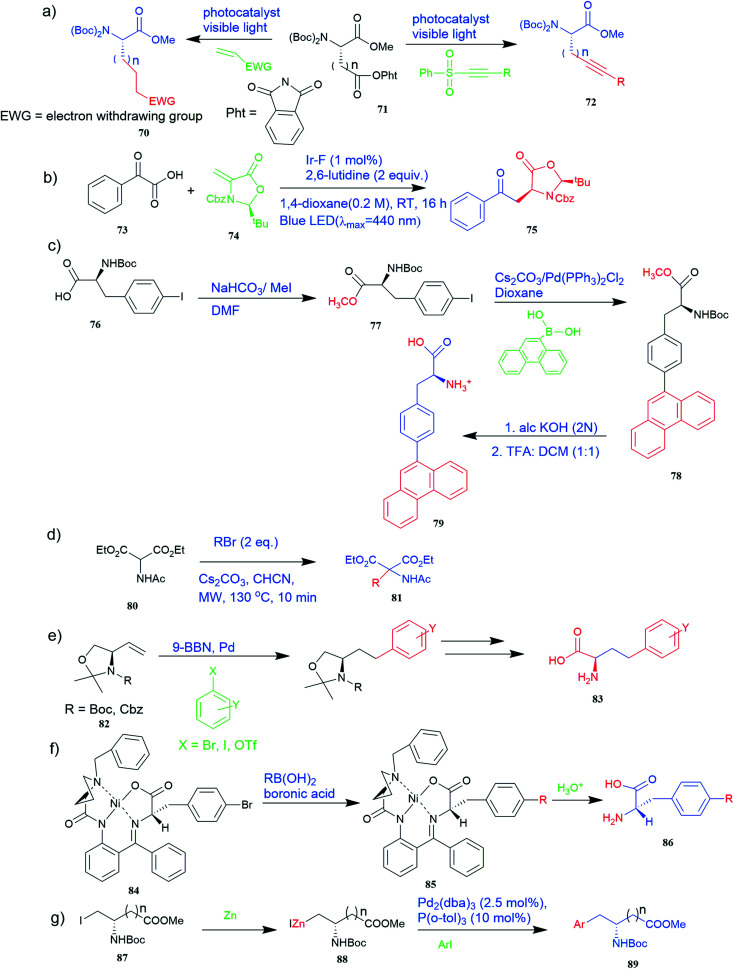
Synthesis of UAAs (a) synthesis of unnatural chiral α-amino acid using visible-light assistance approach (b) light-mediated protocol for the synthesis of UAA *via* the radical decarboxylative process (c) synthesis of fluorescent α-amino acid 79 that emits greenish-blue light (d) synthesis of UAA precursor from diethyl acetamidomalonate 80 (e) synthetic route for various *R*-amino alcohols and *R*-amino acids using Pd-mediated Suzuki cross-coupling reaction. (f) Synthesis of enantiomerically enriched non-protein (*S*)-α-amino acid from Schiff base Ni(ii) complex 84 of the chiral auxiliary (*S*)-BPB. (g) Synthesis of unnatural aspartic and glutamic acid derivative 89.

A method for obtaining unnatural chiral α-amino acids at room temperature using visible light has drawn sufficient interest in protein chemistry. A protocol where two readily available genetically coded proteinogenic amino acids, *i.e.*, l-aspartic acid and glutamic acid derivatives 71 as chiral sources and radical precursors, olefins, alkynyl, and alkenyl sulfones, and 2-isocyanobiphenyl as radical acceptors were used (Fig. 6a). Various unnatural chiral α-amino acids 70 or 72 were synthesized with excellent yields using this method.^51^ A method has also been investigated in which the light-mediated protocol for the synthesis of UAAs *via* radical decarboxylative processes.^52^ In this method, readily available and abundant starting materials such as carboxylic and α-keto acids 73 proceed under very mild reaction conditions and exhibit a high functional group tolerance (Fig. 6b). In addition, it is easy to derivatize radical reaction products so that complex UAAs 75 can be quickly synthesized.

Furthermore, the production of genetically encoded fluorescent α-amino acids that produce greenish-blue light has widespread applications in research, biotechnology, and the pharmaceutical sector. The amino acid 4-phenanthren-9-yl-l-phenylalanine (Phen-AA) 79 that emits greenish-blue light in the visible region has good stability with a 75% quantum yield. As shown in Fig. 6c, its production was initiated with the synthesis of *N*-(*tert*-butoxycarbonyl)-4-iodo-l-phenylalanine methyl ester intermediate (Boc-Phe(4-I)-OMe) 77 where the carboxylate group through methylation of the commercially available *N*-(*tert*-butoxycarbonyl)-4-iodo-l-phenylalanine 76. The next step involved Suzuki–Miyaura cross-coupling reaction which coupled α-amino acids bearing vinyl or aryl halide side-chains with polyaromatic boronic acids and resulted in *N*-(Boc)-4-(9-phenanthracenyl)-l-phenylalanine methyl ester 78 intermediate. De-protection of the methyl and Boc protecting groups of 4 produced the final l-α-amino acid 4-phenanthracen9-yl-l-phenylalanine 79*i.e.* novel fluorescent α-amino acid that emits greenish-blue fluorescence.^53^

#### 2.3.2 Microwave-assisted synthesis

A microwave-assisted method was developed to rapidly prepare racemic UAAs 81 with high yield and purity with a wide range of functional groups tolerance. Here, diethyl acetamidomalonate 80 was used as a precursor (Fig. 5d). These amino acids can potentially be employed in bioconjugation reactions and photochemical transformations.^54^

#### 2.3.3 Cross-coupling reaction

Richard F. Heck (University of Delaware, Newark, DE, USA), Ei-ichi Negishi (Purdue University, West Lafayette, IN, USA), and Akira Suzuki (Hokkaido University, Sapporo, Japan) were awarded the 2010 Nobel Prize in Chemistry for the development of palladium-catalysed cross-coupling reactions in organic synthesis.^55^ This cross-coupling method was found to be applicable in the synthesis of UAAs and many other biochemical reactions. A method for preparing various *R*-amino acids with a variety of unnatural side chains 83 in enantiomeric series from readily available achiral starting materials has been developed where 4-methoxyhomophenylalanine was a compound of interest. The precursors vinyl oxazolidines 82 were obtained through lipase-catalysed kinetic resolutions (Fig. 6e). Critical in this convergent approach was Pd-mediated Suzuki cross-coupling.^56^

Also, a synthetic method for new enantiomerically enriched non-protein (*S*)-α-amino acids 86 was developed to generate new carbon–carbon bonds in the side chain group of the amino acid moiety with high chemical yields and optical purity using the reaction of Suzuki. In this method, as the initial synthon, the Schiff base Ni(ii) complex 84 of the chiral auxiliary (*S*)-BPB (*N*-benzyl proline benzophenone) and 4-bromo-l-phenylalanine amino acid were employed. The final Ni(ii) complexes 85 were decomposed with aqueous HCl, and the amino acids were isolated with excellent enantioselectivities (>99% ee). The chiral auxiliary ligand (*S*)-BPB can be recycled and reused for the synthesis of starting Ni(ii) complex (Fig. 6f).^57^

The Negishi cross-coupling is a powerful C–C bond-forming reaction widely utilized in many areas of organic synthesis. The Negishi cross-couplings were used to synthesize various UAAs like aromatic, heteroaromatic, and complex amino acid derivatives such as aspartic and glutamic acid derivatives 89, aryl glycine derivatives, phenylalanine derivatives, amino acids containing metal-coordinating side chains, β-amino acids, aryl bromides with an iodo serine derivative, naphthyl-appended amino acid, bis(amino acid) derivative, fluoroaromatic amino acid, *p*-(*C*-glucopyranosyl) phenylalanine derivative, cycloalkenyl-protected amino acid derivatives (Fig. 4g and 6g).^58^

Derivatization of natural amino acids including cysteine, threonine, serine, and tyrosine containing heteroatoms such as N, S, O could open the door to synthesizing many possible UAAs. Undoubtedly, novel synthetic methods to construct UAAs will continue to provide new ways for peptidomimetics.

### 2.4 Mutagenesis and incorporation of UAAs

The traditional enzyme engineering approach constitutes the selective substitution of one amino acid by any other 19 natural amino acids (NAAs) to alter the functionalities of enzymes. However, incorporating UAAs provides a better approach to engineering the enzymes for designing efficient biocatalysts that can display novel physicochemical properties and perform biological functions.^59,60^

UAA(s) placed in proteins at strategically selected locations offer a new and fascinating variety of protein research and engineering options.^61^*In vivo* incorporation of UAAs can be carried out in two ways: in site-specific approach and residue-specific approach. The site-specific incorporation uses an exogenously evolved tRNA or synthetase pair, and residue-specific incorporation uses the misacylation of the endogenous tRNA.^60^*In vivo* incorporation methods are marked with many engineering challenges like UAAs transportation across the cell membrane, cytotoxicity, and low incorporation efficiency.^59^

To overcome the challenges of *in vivo* approach, strategies for *in vitro* incorporation of UAAs^62^ have received considerable attention to gain broader specificity and increase efficiency.^59^ The cell-free incorporation of UAAs results in the formation of complex proteins like integral membrane proteins, physiologically toxic proteins, and large protein complexes.^63^ The cell-free incorporation of UAAs can be achieved by global suppression methods and orthogonal translation systems. The former utilizes natural biological mechanisms while later reengineers tRNA, aminoacyl-tRNA synthetase, ribosome, elongation factor, and release factor *via* directed evolution or rational design to incorporate UAAs. The orthogonal translation system is commonly used because of its effectiveness towards site-specific UAAs incorporation in comparison to the global suppression method.^59^ Other ways to incorporate UAAs include solid-phase synthesis and chemical ligation methods. Solid-phase synthesis allows the direct transformation of peptides' side-chains into novel peptides on the solid support. Here, the accumulation of side products prevails because of incomplete deprotection or coupling reactions. This drawback is overcome by chemical ligation methods which permits peptide fragments to the couple in aqueous solutions.^64–66^ Chemical alteration of a synthetic fluorophore protein after translation and the use of chemically mis-attached tRNAs suppressors to insert UAAs are the most straightforward methods. These methods, however, give limited yields of proteins and are restricted to readily available protein locations.^45^ The incorporation of UAAs *via* codon reassignment offers novel chemical and biological functions to translated products.^67^ The amber stop codon (UAG), showing a low level of amino acids misincorporation, is most commonly targeted to reassign and incorporate UAAs during translation in comparison to opal (UGA) and ochre (UAA) stop codons.^63,67^ The UAA mutagenesis was found to enhance the efficiency and regioselectivity of CYP P450 oxidation catalysts.^68^ The detailed mechanism on UAA incorporation is well explained by Young and Schultz,^2^ and Lang and Chin.^66^

## 3. Applications of UAAs

### 3.1 Catalytic functions

Enzymes are potent bio-catalysts that have participated in promoting chemical reactions with remarkable efficiency and selectivity. The effect of incorporating UAAs into enzymes with potential applications in biocatalysis has recently received much attention. Incorporating UAAs into target proteins can improve enzyme activity^69^ and increase the stability of proteins under harsh conditions, such as the resistance to organic solvents.^70^ There are different methods for incorporating UAAs into target proteins with potential biocatalysis applications. One technique is a coupling methodology, which was utilized to design industrially relevant transaminase (ω-TA) since ω-TA shows promising activity in producing optically pure amine compounds.^70^

Furthermore, incorporating UAAs, such as replacing methionine with norleucine, into enzymes may protect proteins from methionine oxidation. This approach is useful for biocatalysts that become inactive in oxidative conditions or require oxidizing substrates or cofactors.^17^ Another method was genetic code expansion (GCE) technology that has been used to introduce small labelling sites in the form of uniquely reactive ncAAs in a target protein. Low incorporation efficiency of UAAs and high background fluorescence limit have super-resolution microscopy (SRM) applications. SRM benefits immensely from the ability to mount photographic fluorescent labels on proteins.^71^ By choosing a sequential allylic C–H amination/vinylic C–H arylation, which began with inexpensive commercially available α-olefins and boronic acids, UAAs precursors were also obtained using Pd(ii)/sulfoxide catalysis. Using coupled enzyme reactions, which are a novel biocatalytic method for synthesizing l-homoalanine from l-threonine consisting of a threonine deaminase (TD) and ω-TA. TD catalyses the dehydration/deamination of l-threonine, asymmetrically converting to l-homoalanine through transamination with benzylamine executed by ω-TA.^72^

*N*-Alkylated-α-amino acids produced *via* enantioselective methods are valuable building blocks for pharmaceutical and fine chemical industries. Therefore, they are treasured and widely investigated. While there are many chemical methods for their synthesis, biocatalytic approaches can give a greener and cleaner alternative to current practices. Alternative processes such as methylation in other proteins and peptides, including *N*-α-methylation, have biological activity of peptides.^73^ For example, cypemycin is a naturally occurring bacterial peptide with post-translational changes, including *N*-α-dimethylation, and it has decisive antibacterial action and *in vitro* efficacy against murine leukemia cells.^74^ Also, *N*-arylated α-amino acids and pyrazolidin-3-ones are widely used in pharmaceuticals and agrochemicals as chiral building blocks. They are biocatalytically produced by utilizing ethylenediamine-*N*,*N*′-disuccinic acid lyase (EDDS lyase) as a biocatalyst. This enzyme has a broad substrate range and high conversions, resulting in high isolated yields and enantiomeric excess of the relevant *N*-arylated aspartic acids.^75^ The use of biocatalyst engineering has greatly aided enzyme discovery and applications in industrial and pharmaceutical applications. The service and advancement of enzyme engineering techniques have grown tremendously in recent years. Importantly, engineering techniques incorporating UAAs successfully produce enzymes with more excellent stability, selectivity, and altered catalytic properties.

In recent decades, applications of enzymes have increased significantly, especially in scientific methodology, pharmaceutical science, food alteration, laundry, biofuel production, agro-industry, and many others. The increasing demand for biocatalysts as a replacement for traditional chemical catalysts has grown progressively. The engineering strategies of enzymes rely on nature's genetically encoded alphabet of twenty canonical amino acids. In the past few years, the emergence of genetic code expansion methods that allow several structurally diverse amino acids to be introduced into the proteins have been observed.^76^

UAA incorporation in enzymes produces enzyme resistance towards temperature and organic solvents, resulting in effective catalytic properties. Various UAAs with different side chains overcome the limitations encountered by NAAs in enzymes, thereby improving their potential applications. The residue-specific method and site-specific method are favoured for producing enzymes with improved and modified functions. Also, it can be done with the coupling of residue-specific and site-specific incorporation methods. Multifunctional green fluorescent proteins (GFP) were constructed through site-specific incorporation of l-3,4-dihydroxyphenylalanine and residue-specific incorporation of (2*S*,4*S*)-4-fluoroproline (4*S*-FP) or l-homopropargylglycine (hpg).^77^ Site-specific methods have attracted considerable attention as proteins or enzymes that bear multi-UAAs display improved functionalities. Interestingly, it has been discovered that integrating two chemically distinct UAAs into GFP by employing two orthogonal pairs in a single expression shows no mutual cross-reactivity and thus can be developed for efficient double labelling. In addition, a highly efficient suppressor plasmid pUltra has been generated, exhibiting higher suppression activity, enabling the efficient suppression of three different amber stop codons in GFP.^78^ In the residue-specific incorporation method, incorporation of 3-fluorotyrosine into ω-transaminase showed a 2 fold higher half-life with enhanced catalytic activity.^79^ Furthermore, the global incorporation of UAAs into β-galactopyranoside resulted in a two-fold increase in *V*_max_ for *ortho*-nitrophenyl-β-d-galactopyranoside at pH 7.0 and a 4–5-fold increase in *V*_max_ toward phenyl-β-d-galactopyranoside at the same pH.^60^ Global substitution using Klentaq DNA polymerase resulted in comparable activity in the same range and similar deoxyribonucleoside triphosphate conversion (dNTP). The addition of (4*R*)-fluoroproline to the DNA polymerase resulted in a fluorinated enzyme that was highly active.^80^

Cytochromes P450 (CYP) are biocatalysts that catalyse the transfer of an oxygen atom from molecular oxygen to an organic substrate upon a donation of electrons by coenzymes, such as NAD(P)H. In P450 BM-3, methionine residue was replaced with the isoteric methionine analogue norleucine to test whether the enzyme stability was hampered by Met oxidation throughout the reaction; a two-fold increase in peroxygenase activity along with a significant reduction in thermal stability has been demonstrated.^81^ Kolev *et al.*^82^ reported P450 BM-3 variant that oxidizes (+)-nootkatone as a representative substrate (since (+)-nootkatone has a various number of different C–H bonds (primary, secondary, tertiary, aromatic) as well as many functional groups such as carbonyl, ester and olefinic group) resulted with the higher turnover number (*k*_cat_) for an engineered CYP on a complex molecule. A five-fold increase in the turnover number (*k*_cat_) by the substitution of Leu75 with *para*-amino phenylalanine (*p*AmF) by stop codon suppression (SCS) in the active site of the enzyme was observed.^82^

Transaminases catalyse the transfer of an amino group between amino acids and α-keto acids, and they act as biocatalysts in the production of optically pure α-amines. The incorporation of *meta*-fluorotyrosine enhanced thermostability and organic solvent tolerance in ω-transaminase by using the selective pressure incorporation (SPI) method.^79^*N*-Terminally truncated version of DNA polymerase I from *Thermus aquaticus* (Klentaq) was generated. Klentaq is a highly thermostable polymerase with no nuclease activity. Replacing 32 proline residues by (4*R*)-fluoroproline in Klentaq DNA polymerase by SPI method was 92% efficient with a highly active fluorinating enzyme.^80^ The substitution of the critical Tyr309 of phosphotriesterase from *Agrobacterium radiobacter* (arPTE) by the SCS method, *i.e.* arPTE variant containing l-(7-hydroxycoumarin-4-yl)ethyl glycine (HCEtG) in place of Tyr results in an eight-fold increase in *k*_cat_ values. Phosphotriesterase hydrolyses organophosphates, such as pesticides with target residues located in the substrate-binding site. The electrostatic repulsion between the HCEtG and the product, which are both negatively charged, contributes to the increased rate-limiting product release step of substrate turnover. The 7-hydroxyl group of l-(7-hydroxycoumarin-4-yl)ethylglycine promoted the hydrolysis of pesticide paraoxon due to interactions between the bacterial phosphotriesterase enzyme and substrate during the Michaelis complex and product release.^83^ Disruption of Asp–His hydrogen bond by replacing the proximal histidine ligand with the unnatural structural analogue *N*-methylhistidine (NMH) by SCS into an engineered ascorbate peroxidase (APX2) achieved significantly increased turnover numbers. Heme peroxidases catalyse a range of oxidative transformations for various biotechnological applications.^84^ Introducing 3-fluorotyrosine to organophosphate hydrolase showed extended optimal pH activity and improved thermal stability at alkaline pH.^85^ Global fluorination of aromatic residues of lipase B from *Candida antarctica* enhanced stability with 4-fluorophenylalanine residue-specific substitution.^86^ Enhanced protein foldability and increased thermostability were shown by the fluorination of phosphotriesterase (PTE) with UAA 4-fluorophenylalanine. Approximately 30% of the enzymes' native structure was maintained.^86^ Replacement of hydrophobic amino acids, such as methionine, proline, and phenylalanine from *Thermoanaerobacter thermohydrosulfuricus* lipase related synthetic analogues like lipase analogues, in a residue-specific manner, resulted in a 25% increase in catalytic activity, substrate tolerance by up to 40%, changes in optimal temperature by up to 20 °C and pH by up to 3.^69^ A change in substrate specificity of the enzyme, *N*-acetylneuraminic acid lyase (NAL) mutants was obtained after incorporating various UAAs, thereby increasing activity towards aldol reactions of erythrose and pyruvate.^87^ Incorporation of UAAs in proteins leads to the mutation of amino acids intimately involved in an enzyme's catalytic mechanism, thereby overcoming challenges such as the substrate or product inhibition, organic solvent tolerance, catalytic efficiency, turnover number, thermal and chemical stability.

### 3.2 Therapeutic applications

UAAs find many applications ranging from being a chiral building block to protein probing to site-specific drug carriers and many pharmacological and therapeutic uses (Tables 1 and 2). UAAs display structural versatility and functional stability so that they can be modified or conjugated to incorporate some target-release drugs. Naphthalene-tripeptides containing alpha-aminoisobutyric acid or alanine have shown various potential uses in combating cancer and bacterial infections.^88^ The UAA *p*-acetyl phenylalanine (*p*AcF) can be integrated with an EGFR targeted, elastin-like polypeptide nanomaterials in the presence of reactive amino acids to produce bio-orthogonal ketone for attachment of doxorubicin, and thus produced nanomaterials are proven to have considerably higher cytotoxicity than non-target controls in several lines of cancer cells.^61^

**Table 1:** Applications of UAAs incorporated enzymes

Enzymes	Name of UAA	Structure of UAA	Characteristics	Incorporation method	References
ω-Transaminase	3-Fluorotyrosine	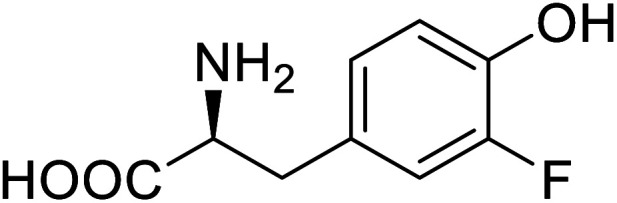	Enhanced catalytic activity and thermostability	Residue-specific incorporation of 4-fluoroproline	79
Organophosphate hydrolase	3-Fluorotyrosine	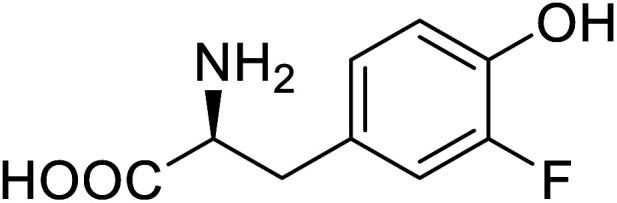	Thermal stability increment at alkaline pH	Residue-specific incorporation	85
P450	Nor-leucine	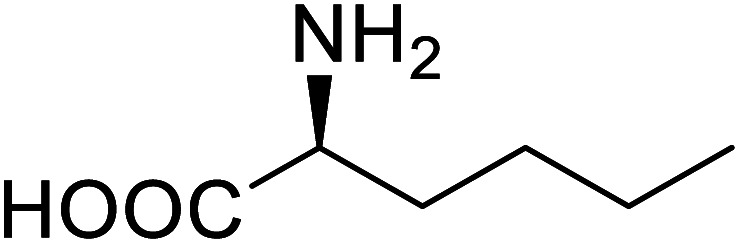	A two-fold increase in peroxygenase activity	Residue-specific incorporation	81
Lipase B	4-Fluorophenylalae	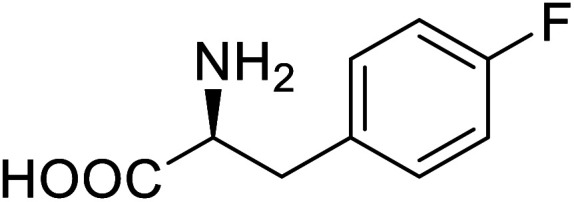	Prolonged the shelf life of lipase activity	Residue-specific incorporation of 4-fluorophenylalanine	86
Phospotriesterase (PTE)	4-Fluorophenylalanine	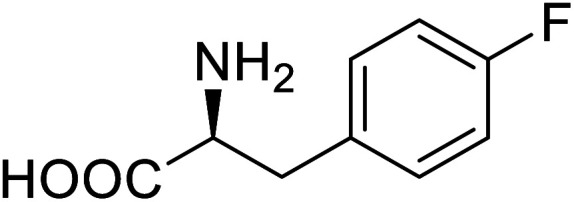	Enhanced protein refolding	Residue-specific incorporation of 4-fluorophenylalanine	115
Green fluorescent protein (GFP)	l-3,4-Dihydroxyphenylalanine	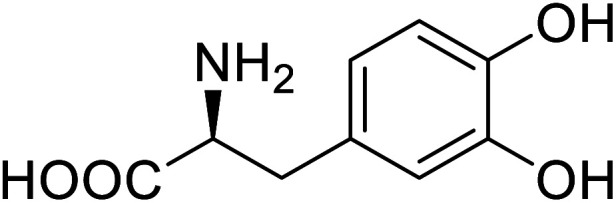	Forming protein–chitosan complexes enhancing stability	Site-specific incorporation of l-DOPA and residue-specific incorporation of 4-fluoroproline	77
Glutathione *S*-transferase (GST)	pNCSF	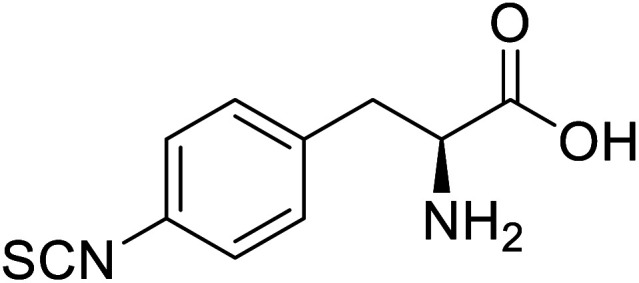	Formation of stable thiourea crosslinks	Site-specific incorporation	83
Lipase	Nor-leucine	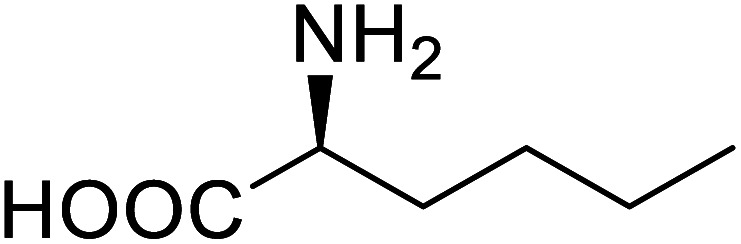	A 10-fold increase in catalytic activity	Residue-specific incorporation	69
Phosphotriester (PTE)	l-(7-Hydroxycoumarin-4-yl)ethylglycine	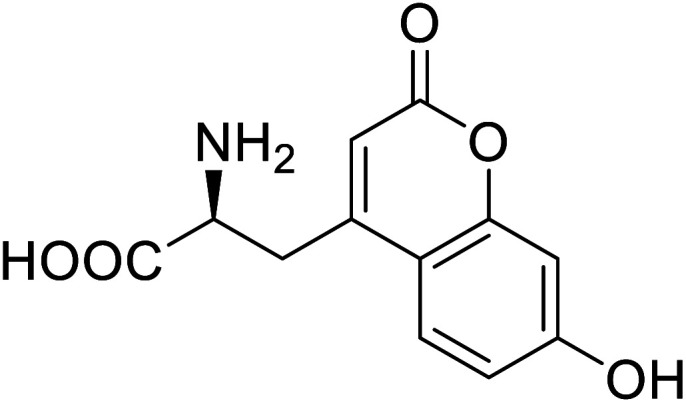	A 8-fold increase in turnover number; promotion of Michaelis complex formation	Site-specific incorporation	83
NAL	2,3-Dihydroxypropyl cysteine	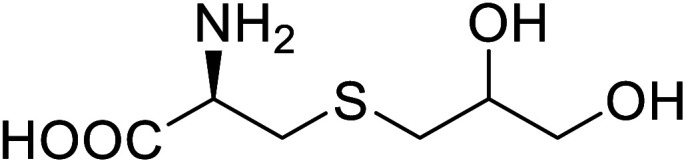	Alters substrate selectivity	Site-specific incorporation	87

**Table 2:** Mode of synthesis, applications, and mode of use of some UAAs

UAA	Structure	Method of synthesis	Application	Mode of use	References
Substituted arylglycine	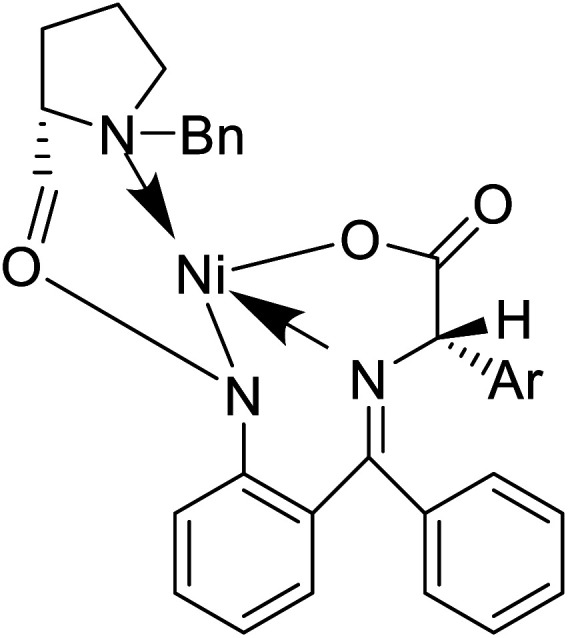	Chemical synthesis	Building blocks in many bioactive compounds and natural products	Assist in drug discovery	46
PrDiAzK	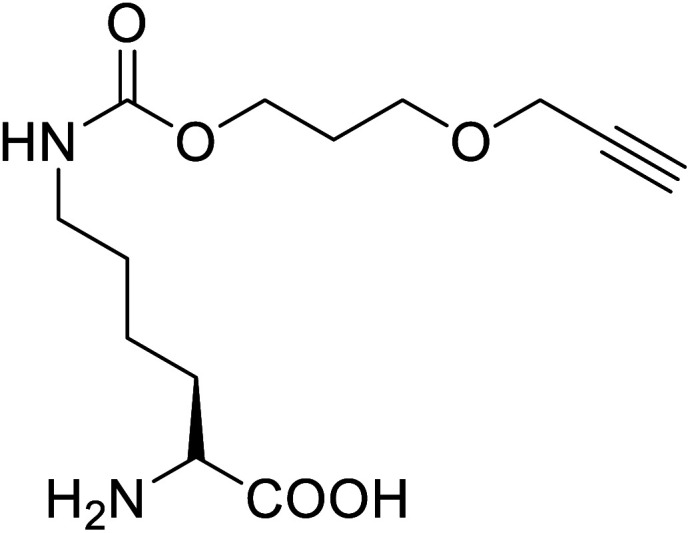	Chemical synthesis	Potential application in the system-wide mapping of protein–protein interaction	Site-specifically incorporated into proteins in both bacterial and mammalian cell culture	47
α-Substituted glutamic acid derivatives	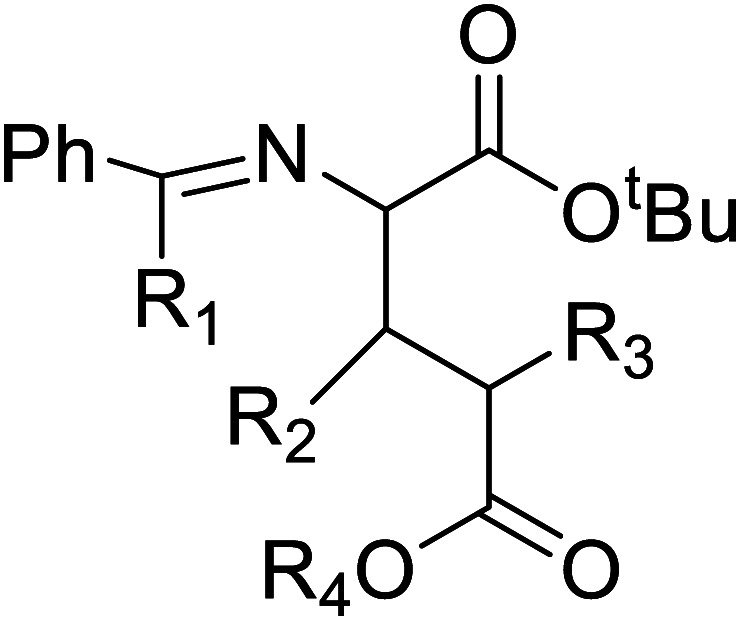	Chemical synthesis	Serves as a new pathway to open new route to biologically active molecules	Shortcut to active biomolecule	124
2-Indolylglycine derivatives	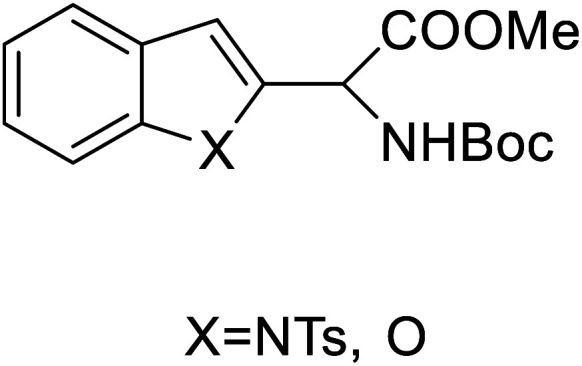	Chemical synthesis	Building blocks in biomolecules	Assist in drug development	34
(*S*)-*N*-(2-Benzoylphenyl)-1-(2-flurobenzyl)-pyrolidine-2-carboxamide	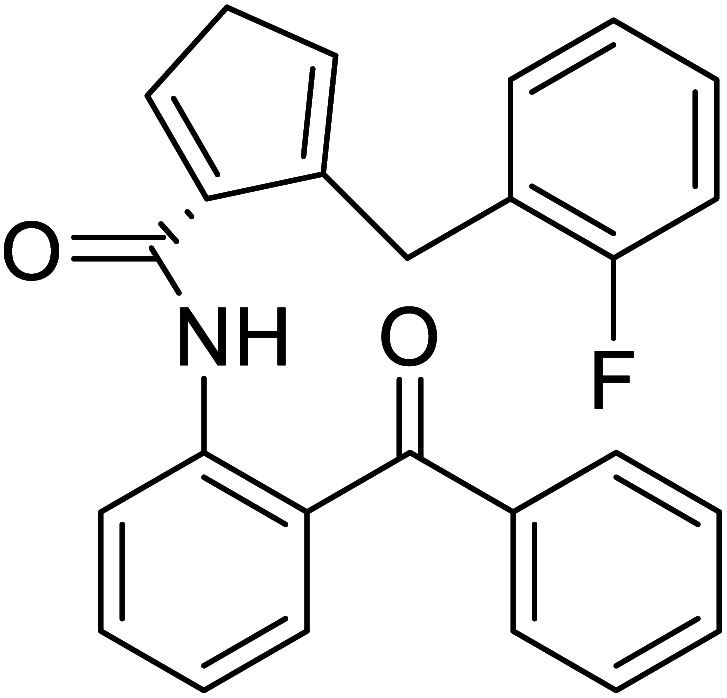	Chemical synthesis	Brings ease and efficiency to stapled peptide research	Peptide stapling	37
4-Phenanthracen-9-yl-l-phenylalanine	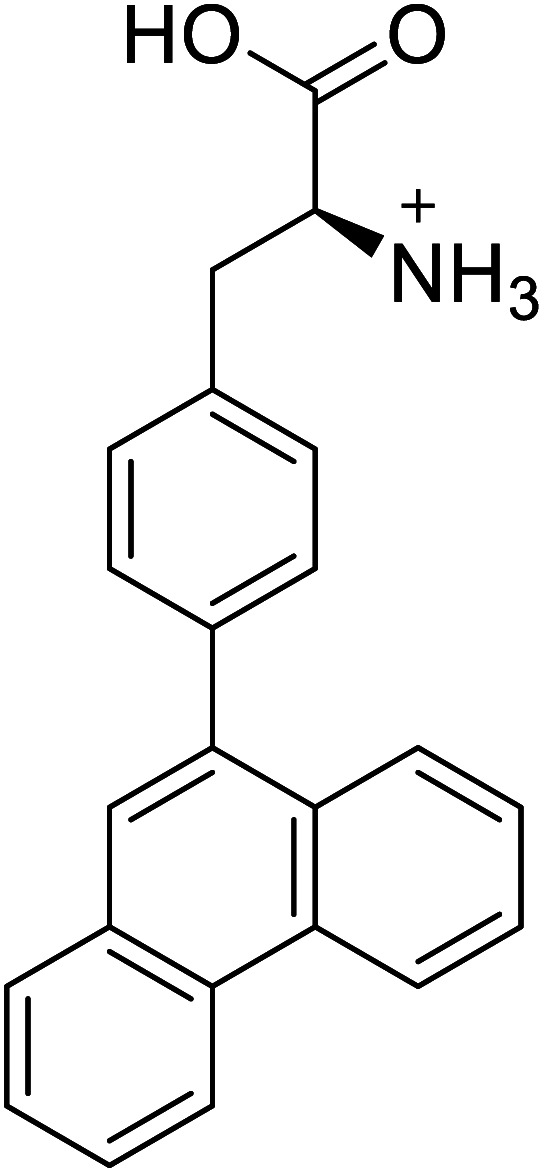	Chemical synthesis	Might find broad application in research, biotechnology, and pharmaceutical industry	Gets into human cells, being visible upon 450 nm laser excitation	53
BN (boron and nitrogen) tryptophan analog	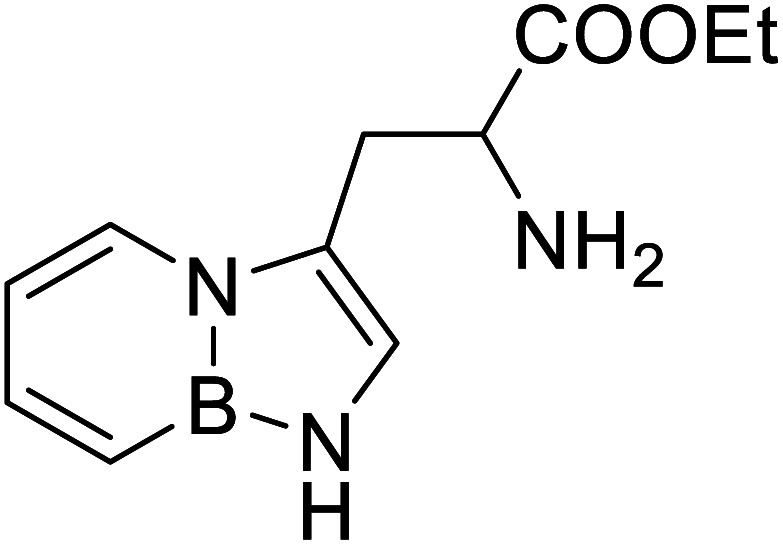	Chemical synthesis	Incorporation with proteins	Employ BN isoterism of arenes in a biological context where tryptophanyl-tRNA synthetase can recognize and azaborine containing amino acid	48
*p*-Acetylphenylalanine	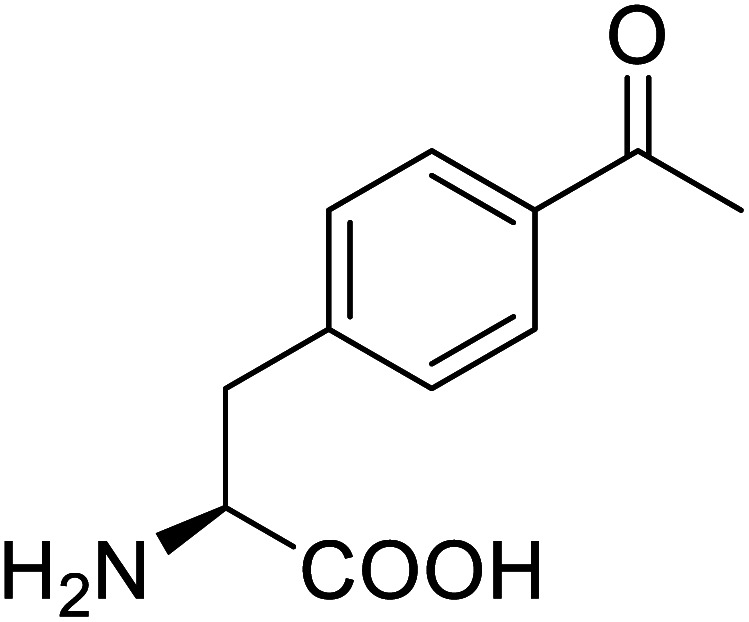	Biological synthesis	Antibody drug conjugates	Part of antibody-drug conjugates	61
γ-Aminobutyric acid (GABA)	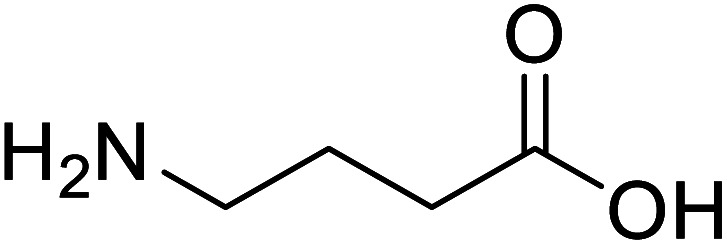	Biological synthesis	In metabolic engineering	To develop engineered strain	14
l-Azidohomoalanine	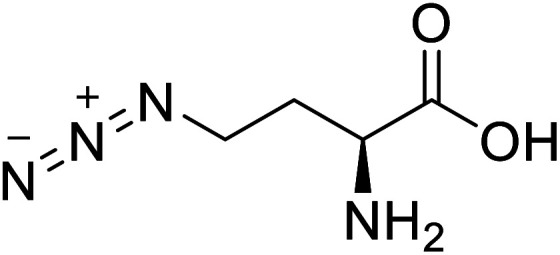	Biological synthesis	Investigate more engineering approaches and metabolic pathways	Biosynthesis of other pharmaceutically valuable UUAs	125
*p*-Azidomethyl-l-phenylalanine	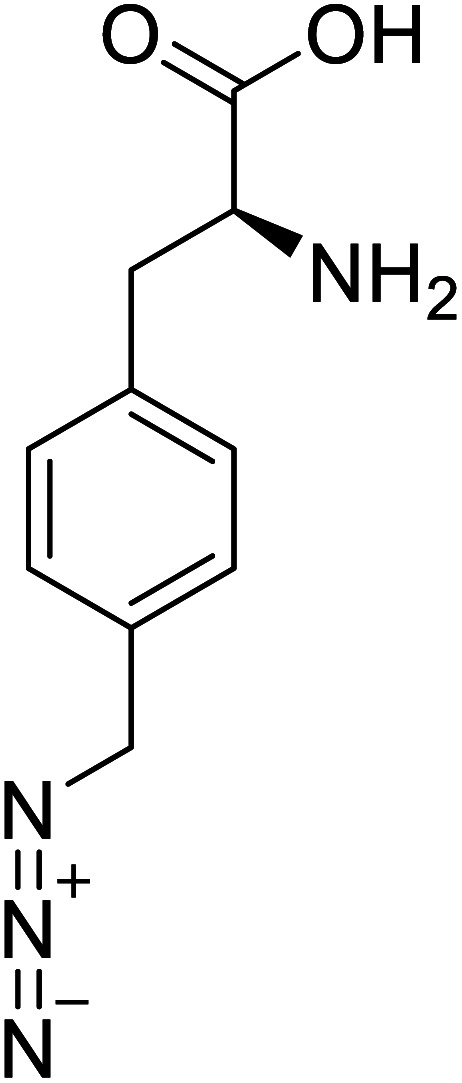	Chemical synthesis	Antibody drug conjugates	Part of antibody-drug conjugates	93
*O*-[^18^F]-Fluoroethyl-l-tyrosine	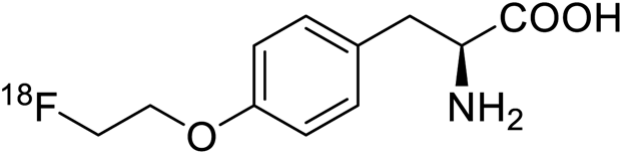	Radiochemical synthesis	Tumor imaging agent for PET	Radiolabeled tracer	74
^18^F-FDOPA	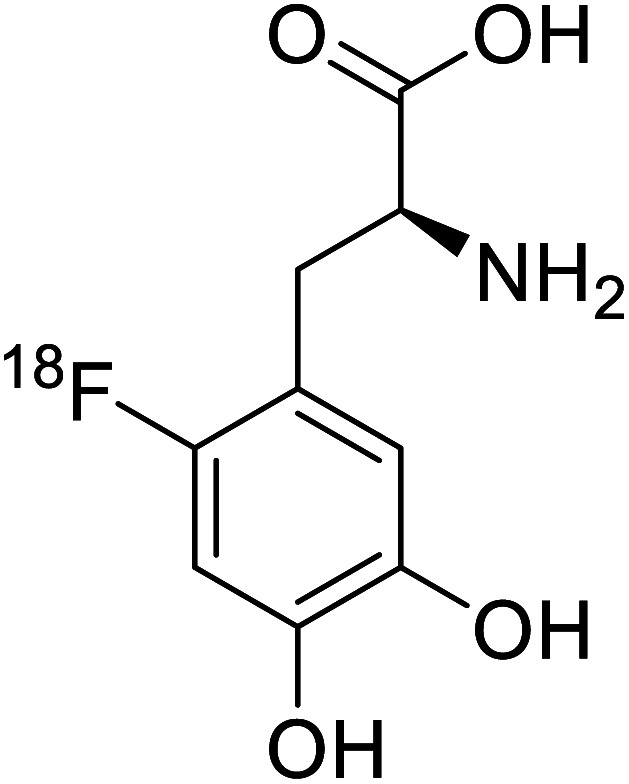	Chemical synthesis	Tumor imaging agent, PET	Radiolabeled tracer	100
^18^F-Fluciclovine	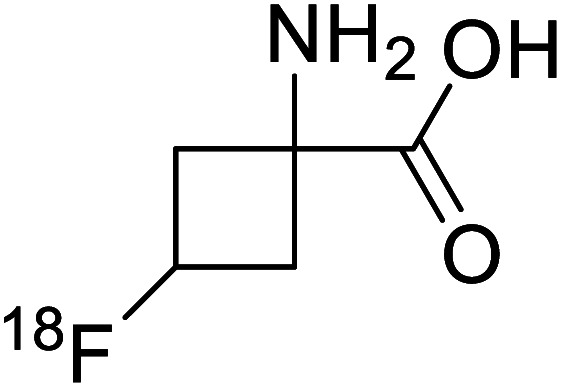	Chemical synthesis	PET tumor imaging agent	Radiolabeled tracer	101
l-(7-Hydroxycoumarin-4-yl)ethylglycine	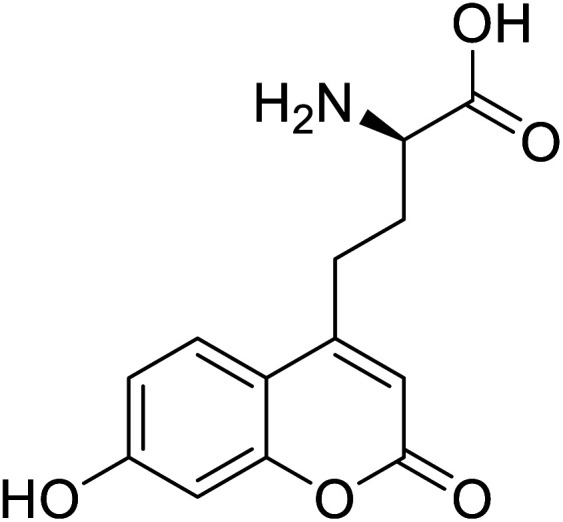	Chemical synthesis	Preparation of fluorescently labeled protein	Incorporated to improve catalytic activity	126
*N*-Acetylated fluorophenylalanine	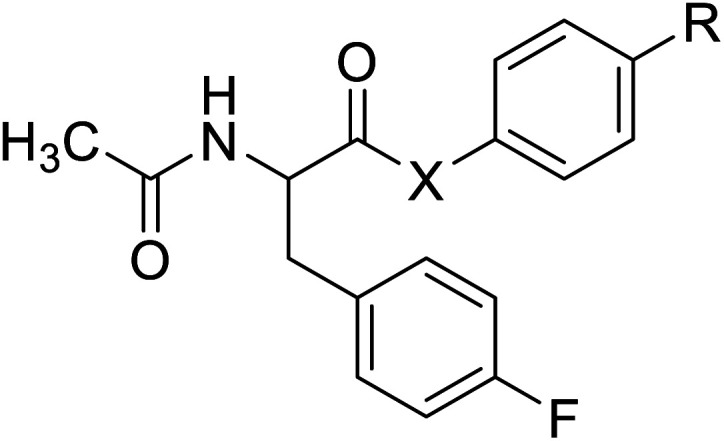	Chemical synthesis	Antiviral, anticancer drug conjugates	Part of antibody-drug conjugates	127
4-Fluoroproline	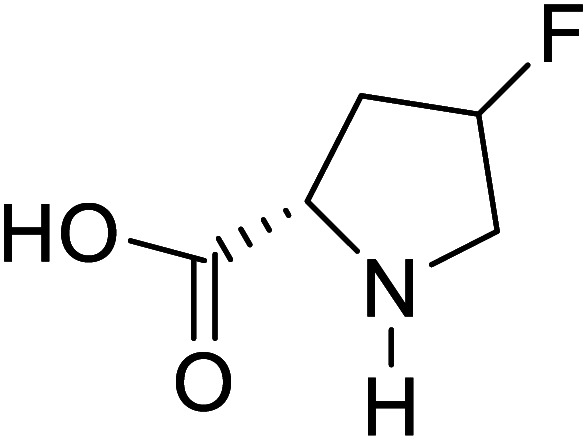	Chemical synthesis	Protein design and engineering	Enhances conformational stability upon proteins	128
Sarcosine (*N*-methyl-α-l-glycine)	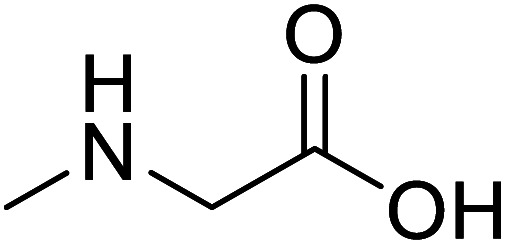	Biocatalytic synthesis	Used as a dietary supplement and as a non-specific glycine transport	Building blocks for the pharmaceutical	73
*N*,*N*-Dimethylglycine	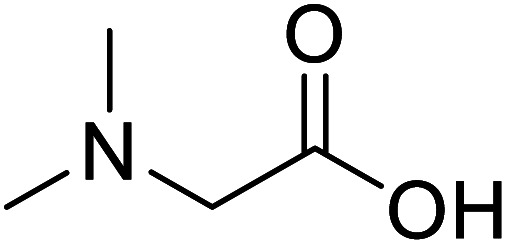	Biocatalytic synthesis	Acts as an athletic performance enhancer, displays anticonvulsant activity inhibitor	Building blocks for the pharmaceutical	73
Betaine (glycine betaine)	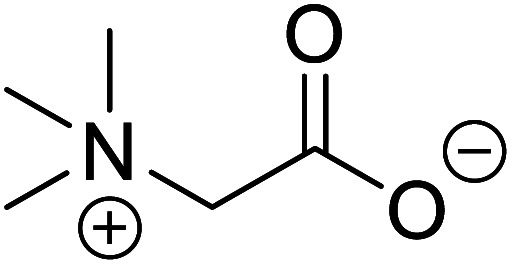	Biocatalytic synthesis	Treatment for homocystinuria and can offer benefits to human health, attenuation of liver injury	Display antioxidant and antihistaminic activity	73

### 3.3 Antibody-drug conjugates

Antibody-drug conjugates (ADCs) have the potential to become the next generation of cancer therapeutics. UAAs are widely used in ADCs and result in better therapeutics due to their unique orthogonal coupling, site-specificity, and reduced toxicity. Among many UAAs, p-acetyl phenylalanine has been widely used in ADCs. Incorporating *p*AcF in human growth hormone (hGH) at specific locations allowed site-specific polyethylene glycol (PEG) conjugation to produce variants of hGH that are more effective and safer than hGh produced by mammalian cells as demonstrated in clinical trials of GH deficient adults.^89^ Also, when *p*-AcF was site-specifically incorporated into *anti*-CD11a IgG and then conjugated into aminooxy-modified LXR agonists, an ADC that demonstrated three-fold higher activation in humans THP-1 monocyte/macrophage cells *in vitro* than conventional LXR agonists have resulted.^90^ Thus produced ADC could be a better therapeutic agent for atherosclerosis. Following a similar strategy, conjugation of human antibody CD11 to highly potent PDE4 inhibitors resulted in a novel ADC with better therapeutic potential as an anti-inflammatory drug due to improved drug specificity.^91^

Genetically encoded ncAA with orthogonal chemical reactivity are used for the synthesis of ADCs. Such produced ADCs have precise control over linkage site and stoichiometry, facilitating optimization of ADCs as therapeutic carriers and coupling agents.^92^ Another ADC with greater effect *in vitro* cell cytotoxic assays was produced through strain-promoted alkyne–azide cycloaddition (SPACC) copper-free click chemistry when unnatural amino acid, *para*-azidomethyl-l-phenylalanine (*p*AmF), was site-specifically incorporated, facilitating almost complete conjugation of DBCO-PEG-MMEF drug to the tumour-specific Her2-binding IgG trastuzumab.^93^

These days the main challenge in applying peptide therapeutics is their relatively short plasma half-life and the corresponding *in vivo* stability.^94^ A new molecule is thus introduced, called cell-penetrating peptides (CPPs), a short-chain of 5–30 amino acid residues that are widely used for the effective intracellular transportation of huge varieties of biologically active drug molecules, drug delivery vectors as well as cargos *via* biological membranes.^95^ CPPs can be a crucial aspect of therapeutic vaccines and drugs. A bio-reducible CPP, a branched m-R9 (B-mR9), was synthesized from nona-arginine (mR9) modification using disulfide bonds whose unusual polyplex B-mR9/siVEGF efficiently inhibited tumour sites and tumour growth as well as showed high accumulation and strong retention in comparison to other groups.^96^ Some of the restricted secondary structures of the peptides due to the presence of UAAs make them enzyme resistant compared to those containing natural amino acids.^60^ This greatly benefits the strength of CPPs.

### 3.4 Imaging agent

Positron emission tomography (PET) is an imaging technique where radioactive tracers scan metabolic and physiological activities. The UAA *O*-[^18^F]-fluoromethyl-l-tyrosine acts as a superior PET imaging agent for tumour detection and cancer imaging by amino acid transport^97^ as well as for differentiating tumour and inflamed tissues.^98^ This method is found more effective than conventional imaging techniques such as MRI. The [^18^F]-fluoro-ethyl-tyrosine PET shows high accuracy in diagnosing brain tumour patients and glioma in early stages, and it is also efficient in differentiating low grade from high-grade tumours.^99^ Radiolabeled amino acids are quickly becoming a choice as PET tracers because normal brain tissues poorly absorb such radiolabeled amino acids. Thus, brain tumours could be differentiated from normal brain tissues with high contrast.

^18^F-FDOPA(6-[^18^F]-fluoro-l-3,4-dihydroxyphenylalanine) based PET/CT imaging was found to be very sensitive to post-treatment diagnosis of head and neck paragangliomas (HNPGLs),^100^ and diagnosis of pheochromocytoma-paraganglioma induced tumour.^99^ These treatments are found to be more effective than conventional MRI/CT by many such experimental trials. It could be a useful tool in the diagnosis of many neuroendocrine tumours. ^18^F-Fluciclovine (*anti*-1-amino-3-^18^F-fluorocyclobutane-1-carboxylic acid (FACBC)) PET/CT is an effective tool for imaging breast cancers even malignant ones such as invasive ductal carcinoma (IDC) and invasive lobular carcinoma (ILC), and also detection of extra-axillary nodular metastases.^101^ It also distinguishes between benign tissues and malignant tumours.^102,103^ PET/CT scans have been proved to be up-and-coming tools for imaging and a more precise diagnosis of nodal disorders in preclinical investigations. These studies imply that many of the flaws in traditional diagnostic approaches may be overcome.

### 3.5 Localization of proteins

Fluorescent UAAs have opened an efficient way of studying protein functions, conformational changes, and their localizations. Unnatural fluorescent amino acids (FIAAs) can label macromolecules fluorescently without altering their biomolecular properties, thereby facilitating the study of complex molecular processes.^104^

A coumarin-based fluorescent amino acid (*S*)-1-carboxy-3-(7-hydroxy-2-oxo-2*H*-chromen-4-yl)propan-1-aminium (CouAA) was incorporated into *E. coli* FtsZ using an evolved Mj tRNA^Tyr^/TyrRS pair for the visualization of *in vivo* localization of particular proteins.^105^ Tryptophan is the most widely used amino acid as a fluorescent reporter for studying protein dynamics and functions. It has high sensitivity towards the electrostatic environment and greater quantum field.^106^ Various modifications are performed on tryptophan to synthesize more suitable and better-functioning fluorophores. A deep blue coloured tryptophan analogue, beta-(1-azulenyl)-l-alanine, having characteristic fluorescent and photophysical properties, was synthesized using Neighisi coupling. Upon incorporating this UAA into argyrin C, the group showed great potential in the localization and visualization of targeted proteins.^107^ Another tryptophan analogue, 4CN-Trp being a small fluorescent UAA possessing unique photochemical properties, is a potential candidate for its use *in vitro and in vivo* spectroscopic and microscopic studies of proteins.^108^

### 3.6 Antimicrobial peptides

Antimicrobial peptides (AMP) are amphipathic and cationic molecules with 10–50 amino acid residues that constitute key parts of the innate immune system and are considered potential alternatives to antibiotics as new therapeutic agents. To address these restrictions, certain changes have been made, and these include the use of peptidomimetic backbones,^109^ technical modification^110^ truncations from parental peptides, and incorporation of UAAs.^109,111^

Due to dominating activity that is ineffective and low serum stability, systematic Cbf-14 modification by incorporating UAAs (ornithine [Orn], norleucine [Ile], and d-amino acids) results in a positively charged peptide mutant Cbf-14-2 with similar stability, negligible toxicity, and superior antimicrobial activities against penicillin-resistant bacteria both *in vivo*/*in vitro*.^112^ An *in vitro* study hinted toward a selective anti-cancer effect of Cbf-K16 against human non-small cell lung carcinoma H460 cells.^113^ Antibacterial peptides (ABPs) incorporated with UAAs (uABPs) are essential for host defence against microbial infections.

UAAs can be utilized to improve bio-redox processes. For example, the abiotic nicotinamide dinucleotide flucytosine cofactor can assist in identifying protein structures and modifying them after translation.^114^ A novel medication type, which can replace or be an alternative to treatments now utilized, has been found by UAAs synthesizers with its unique advantages. Further therapeutic research uses UAAs to aid unriddle emerging and unsolved pathogenic diseases.

### 3.7 Probes for changes in protein conformation

The ability to insert UAAs into proteins on a site-specific basis has significantly impacted protein engineering. In 2020, according to Dangerfield, although bacteriophage T7 DNA polymerase undergoes significant substrate-induced conformational changes that are considered to account for high replication fidelity, previous studies were hampered by mutations necessary to create a Cys-lite variation for site-specific fluorescence labelling. Dangerfield and Johnson employed orthogonal amber suppression machinery in *E. coli* to optimize the direct integration of fluorescent artificial amino acid (7-hydroxy-4-coumarin-yl)-ethylglycine.^116^ While screening a large number of random natural variants, site-directed incorporation of l-(7-hydroxycoumarin-4-yl)ethylglycine UAA into a bacterial phosphotriesterase improved catalytic activity significantly as compared to any natural mutation.^117^ Several UAAs have been introduced at a similar range of sites in dozens of proteins, demonstrating the generality of the nonsense suppression method. The majority of applications have used caged side chains, where an *o*-nitrobenzyl group shields a heteroatom. Photolysis eliminates the nitrobenzyl after a UAA is incorporated into a protein with caged side chains, revealing the previously caged functionality.^118^ The use of genetic code expansion to site-specifically incorporate UAAs into POIs is a potent option. The amber codon suppression approach can be used to integrate various functional groups such as azides, alkynes, alkenes, and tetrazines, which are then changed using appropriate chemicals. For example, integrated UAAs azides or alkynes can conduct a copper-catalysed azide–alkyne cycloaddition (CuAAC).^119^ When calcium binds to calmodulin protein (CaM), conformational changes occur, allowing Ca^2+^/CaM to recognize and bind a variety of target proteins with high affinity. Incorporation of *p*-azido-phenylalanine (AzF) in various positions of CaM, followed by infrared (IR)-spectroscopy of the azido stretching vibration. It distinguishes between distinct binding motifs based on the azido stretching modes spectrum properties.^120^ The use of vibrational probes containing unnatural amino acids with distinct altered side chains (*e.g.*, nitrile, thiocyanate, azide) has substantially increased the application of vibrational spectroscopies to investigate local dynamics and conformational changes with residue-specific resolution. The stretching vibrations of the ester carbonyl side chain of numbers of UAAs is not only localized, but its frequency also varies linearly with an electrostatic field in both hydrogen bonding and non-hydrogen bonding environments.^121^ The addition of unnatural moieties, including fluorophores, affinity labels, spin-label probes, isotope, and bioorthogonal functional groups, introduced to proteins for several *in vitro* and *in vivo* processes and potential applications of such fusion proteins in molecular imaging.^122^ Genetic incorporation of UAA such as ketone-bearing amino acid *p*-acetyl phenylalanine (*p*AcPhe) into recombinantly expressed proteins has provided an elegant alternative to cysteine-based labelling methods where traditional thiol-based SDSL is not feasible.^123^

## 4. Conclusions, limitations, and future perspectives

UAAs are non-proteinogenic amino acids that can be found in nature or can be synthesized chemically. Admittedly, the biological synthesis of UAAs has been economically feasible for large-scale production than the chemical synthesis. They are commonly utilized as blocks of chiral construction. Though d- or aberrant amino acids can be utilized to replace l-amino acids in physiologically active peptides, too much modification or replacement can cause cytotoxicity, immunogenicity, and other problems.

Whereas UAA incorporation into proteins, being a powerful tool to modify the properties of proteins, this approach is becoming more attractive for biotechnological uses. Some biotechnological tools include molecular imaging probes, protein–protein interaction probes, therapeutic proteins, enzymes, fluorescent probes, biocontainment, biomaterials, and biocatalysts with novel functional and structural properties which can be prepared *via* UAA incorporation into proteins. Although the results obtained are eye-catching and promising, the potential is still far from achieved. It has been demonstrated that methyl ester form of UAAs against free acid form showed much better yield; the improved yield is possibly due to the improved membrane permeability of the methyl ester forms of the UAAs to *E. coli* cells as compared to the free acid forms, even though the same tRNA synthetases and *E. coli* strains were used for incorporation.^129^ A study in 2020 optimized the direct incorporation of a fluorescent UAA (7-hydroxy-4-coumarin-yl)-ethyl glycine, where MS methods confirm that the UAA is introduced at only one site and that there is limited precedent.^116^

The biochemical implications of altering the cellular translation machinery, as employed in GCE-based tagging, have yet to be extensively explored. First, cells were not examined or had an impact on ribosomal function in the cellular response to an exogene tRNA/tRNA synthetase combination. Second, the physiological effects of protein reading induced by UAAs are not yet fully examined in TAG endogenous codons.^130^ The integration of UAAs into proteins has been an area of active interest for several fundamental and applicable research sciences. Despite the various types of synthetic UAAs, due to the limited number of studies available, further studies are highly recommended. In the field of amino acids and peptide/protein chemistry, directing groups (*e.g.*, 8-aminoquinoline) and protecting groups (*e.g.*, Phth) are uncommon. The introduction and removal of these protecting and directing groups necessitate additional steps, lowering the atom and phase efficiency of the modification process. Even with the aforementioned current challenges, we predict that C–H functionalization would become a cost-effective solution for synthesizing many UAAs as the repertoire of the C–H functionalization technique grows.

*E. coli* is primarily utilized for large-scale protein synthesis. On the other hand, this prokaryotic workhorse is less frequent and incapable of producing post-translational modifications, including glycosylation, ubiquitination, phosphorylation, and other eukaryotic and proteolytic protein maturation. Furthermore, eukaryotic and mammalian cell systems are prone to contamination, frequently requiring specific growth sites and bypassing or disabling glycosylation systems to produce humanized therapeutic proteins without introducing additional factors that may induce immunogenicity. Both stationary phase biology and post-translational modifications in prokaryotic systems are still active research areas with many unanswered questions. Synthesizing appropriate UAAs and assembling UAAs and scaffolds to produce functional catalysts are distinct synthetic problems that cannot be overlooked or obscured by similar research aimed at uses other than catalysis. The absence of library techniques for iterative scaffold optimization is perhaps the biggest roadblock to creating hybrid catalysts that use UAAs.^131^

When acidic conditions were adopted for deprotection at the oxazolidinone centre, 96 percent of the free amino acid was produced. In addition, oxazolidinone species I and II may be easily derived to get more complex UAAs. Control data indicates that to introduce alkylation reactions under normal circumstances, there should not be the presence of any light or photocatalyst.^52^ Recent primary and secondary C(sp^3^)–H bond functionalization processes have selectively changed natural amino acids and peptides. Sequential C–H activation and C–X (X = C, B, O, N, F, *etc.*) bond formation reactions have successfully changed the side chains of amino acids. Even though substantial progress has been made in this area, there are still several obstacles to overcome. The reactivity of unactivated C(sp^3^)–H bonds, especially secondary C(sp^3^)–H bonds, is still low, which can be overcome by strategically developing new ligands and directing groups.^132^ ncAA-mediated methods broaden the range of things identified and generated, such as conjugates, vaccinations, and cell-based treatments. It is also becoming clear how important it is to apply medicinal chemistry ideas to bigger proteins, *i.e.*, protein medicinal chemistry. Given the potency of channels for discovering therapies that employ just canonical amino acids, granting them full access to the chemical cabinet would very definitely result in totally new therapeutic groupings.^131^

The asymmetric synthesis of UAAs is a significant step to incorporate optically pure UAAs in natural proteins during the reprogramming of natural proteins by using UAAs. Therefore, an extensive study on enantioselective, catalytic, and chiral pool synthesis of optically pure UAAs has been reported in recent years.^43–51^ Further study in the design of novel catalysts and chiral auxiliaries, as well as proper selection of chiral precursors for chiral pool synthesis, could enhance the synthesis of chiral UAAs with an excellent enantiomeric excess (ee) and high yield. Consideration of the green chemistry approach towards the asymmetric synthesis of UAAs is highly recommended.

There are a limited number of UAAs in the current defined metabolic pathways, and in some cases, essential pieces of information are missing, including the enzymes involved. During the biosynthesis process, some UAAs inhibit the growth of chassis strain and may cause trouble in fermentation processes. Hence, the prospect needs the involvement of crucial enzyme representation and clear metabolic pathways. For balanced metabolism and increased resistance, more chassis strain is needed to be metabolically engineered or screened. After examining secondary structures of polypeptides containing proteinogenic amino acid residues, it isn't easy to anticipate the conformation of contemporary, non-natural polypeptide materials based on their side-chain structures. To elucidate their effects on secondary structures, fundamental understandings of side-chain interactions, such as charge, H-bonding, hydrophobic, and dipole–dipole interactions, are needed. Ultimately, synthetic polypeptides can be used as standalone therapeutics in addition to using polypeptide products as nano-carriers. Glatiramer acetate (GA), a statistical copolypeptide with four natural amino acid residues, is one noteworthy example.^133^

The full promise of protein medicinal chemistry and other ncAA applications in biotherapeutics will be realized only when high-performance ncAA integration systems in bacterial and eukaryotic expression systems are available. Given the strength of biological platforms using only canonical amino acids, full access to the chemical cabinet provides these platforms with completely new therapeutic classes.^134^ Future research should concentrate on reaction designation, enzyme property enhancement, and process optimization to develop commercially feasible platforms for derivatizing amino acids. Biocatalysts will likely be used to manufacture additional fine chemicals from amino acids on a commercial scale in the future.^135^ Scopes of UAAs in biotechnology, synthetic biology, and traditional tools should be upgraded by well-designed algorithms and computer-aided enzyme/protein molecular prediction, which supports the practical implementation of UAAs and helps to design different artificial enzymes.

## Author contributions

A. Adhikari, B. R. Bhattarai, A. Aryal, N. Thapa, P. KC, Ashma Adhikari, and S. Maharjan reviewed the literature. P. B. Chanda, and B. P. Regmi executed critical revision. A. Adhikari, B. R. Bhattarai, and N. Parajuli drafted the manuscript. All authors accepted the submission for publication.

## Conflicts of interest

There are no conflicts to declare.

## Supplementary Material
